# LGBTQ+ Perspectives on Conducting Genomic Research on Sexual Orientation and Gender Identity

**DOI:** 10.1007/s10519-022-10105-y

**Published:** 2022-05-26

**Authors:** Catherine Hammack-Aviran, Ayden Eilmus, Carolyn Diehl, Keanan Gabriel Gottlieb, Gilbert Gonzales, Lea K. Davis, Ellen Wright Clayton

**Affiliations:** 1 Center for Biomedical Ethics and Society, Vanderbilt University Medical Center, 2525 West End Ave., Suite 400, Nashville, TN 37203 USA; 2 College of Arts and Sciences, Vanderbilt University, Nashville, TN USA; 3 Program for LGBTQ Health, Vanderbilt University Medical Center, Nashville, TN USA; 4 Department of Medicine, Health and Society, Vanderbilt University, Nashville, TN USA; 5 Vanderbilt Genetics Institute, Vanderbilt University Medical Center, Nashville, TN USA

**Keywords:** LGBTQ+ health, Sexual orientation and gender identity, Qualitative research, Attitudes about genomics research

## Abstract

We conducted in-depth, semi-structured interviews with LGBTQ+-identified individuals (n = 31) to explore the range of LGBTQ+ perspectives on genomic research using either sexual orientation or gender identity (SOGI) data. Most interviewees presumed that research would *confirm* genetic contributions to sexual orientation and gender identity. Primary hopes for such confirmation included validating LGBTQ+ identities, improved access to and quality of healthcare and other resources, and increased acceptance in familial, socio-cultural, and political environments. Areas of concern included threats of pathologizing and medicalizing LGBTQ+ identities and experiences, undermining reproductive rights, gatekeeping of health or social systems, and malicious testing or misuse of genetic results, particularly for LGBTQ+ youth. Overall, interviewees were divided on the acceptability of genomic research investigating genetic contributions to sexual orientation and gender identity. Participants emphasized researchers’ ethical obligations to LGBTQ+ individuals and endorsed engagement with LGBTQ+ communities throughout *all* aspects of genomic research using SOGI data.

## Introduction

Sexual orientation describe a person’s sexual identities, sexual attractions, and sexual behaviors with the same or different-sex partners, while gender identity, gender expression, and gender presentation (hereinafter “gender identity”) describe whether a person is a man, woman, neither, or other gender(s) (e.g., non-binary, genderqueer, or gender nonconforming). Like many complex behavioral traits (Fisher et al. 2018), sexual orientation and gender identity are highly heritable and are hypothesized to be influenced in part by genetic variants (Polderman et al. 2018), which researchers have sought to uncover over the years. Among numerous investigations throughout the 1990s (Bailey and Pillard 1991, 1995; Pattatucci and Hamer 1995; Veniegas and Conley 2000), one particularly notable study reported that a region at Xq28 was associated with homosexuality in cisgender men (Hamer et al. 1993), eliciting a firestorm of criticism (Haynes 1995; Marshall 1995; McGuire 1995; Ordover 1996; Risch et al. 1993).

Following a decades-long lull in genomic research to identify genetic variants that contribute to sexual orientation and gender identity, recent efforts to collect and analyze data on millions of people to understand the complex contributions of genetic variation and individual environments towards health outcomes and other characteristics has spurred a resurgence of interest in this topic (Mapes et al. 2020). Thus, in 2019, Ganna et al., whose data came from the UK Biobank and 23andMe, reported that using data from the UK Biobank they had identified a number of genes associated with males who had had a same-sex experience, (Ganna et al. 2019) which led to extended debate (Ganna et al. 2021; Hamer et al. 2021). Since that time, three other groups have published research identifying genetic contributions to male sexual orientation (Hu et al. 2021a; b; Sanders et al. 2021; Zietsch et al. 2021).

Yet using genomic tools to explore complex behavioral traits is not without controversy and extend well beyond sexual orientation and gender identity. For instance, a recent scoping review regarding using genomic approaches to explore psychiatric disorders identified 22 ethical, legal, or social issues, most commonly including privacy, stigma, psychological harm, and discrimination, concerns that have often led to “recommendations to limit the use” of those approaches (Iitis et al. 2021). Similarly, over the years, several scholars have argued that the risks of research to understand genetic contributions to sexual orientation and gender identity, such as use of the results for discrimination (Schüklenk et al. 1998; Stein 2007), may outweigh the benefits—concerns that continue to the present (Diamond and Rosky 2016; Savulescu et al. 2021; Smilan 2020). Others, however, have urged that research could be conducted ethically. (Joslyn and Haider‐Markel 2016; Murphy 1997; Polderman et al. 2018).

Meanwhile, there is growing consensus that perspectives and insights from individuals and groups who would be most directly affected by or involved in research—particularly those who are marginalized, vulnerable, or historically underrepresented in or excluded from research—are essential to the ethical and scientific integrity of study design, implementation, and dissemination (Erves et al. 2017; Iitis et al. 2021; Joosten et al. 2015). Prominent examples of engagement include the *All of Us* project (Mapes et al. 2020), the Center for the Ethics of Indigenous Genomic Research (Hiratsuka et al. 2020; Wagner et al. 2020), and the International HapMap Project (International HapMap Consortium 2004). Indeed, the International Gender Diversity Genomics Consortium conducted community engagement studios as part of their work (Polderman et al. 2018).

Yet despite calls for engagement with individuals who are LGBTQ+ to learn about their perspectives about research to understand genetic contributions to sexual orientation and gender identity, recent data are limited. One online survey, which oversampled LGBTQ+ individuals, demonstrated that those with generally favorable attitudes toward LGBTQ+ people were more likely to endorse research investigating genetic contributions to sexual orientation and gender identity, particularly if it revealed a genetic link to gender identity, although those who were younger, were more tolerant of sexual and gender minorities, and had more knowledge about genetics were more likely to worry that the public would misinterpret genetic research on sexual orientation and gender identity (Thomas et al. 2020). Qualitative research can complement findings of survey-based research in important ways because the interviewer can probe issues in greater depth and follow up on participants’ responses. Thus, Rajkovic and colleagues recently reported the results of semi-structured interviews of 18 transgender and gender diverse (TGD) individuals about trans-associated genetic research. They reported that even those who felt that such research could be beneficial nonetheless were concerned that the results could be misused. Notably, these respondents urged investigators to engage with the TGD community when conducting research on genetic variants associated with TGD identities (Rajkovic et al. 2021).

To further inform the ethical use of data about sexual orientation and gender identity (SOGI) in genomic research, we conducted in-depth semi-structured interviews with self-identifying LGBTQ+ individuals to elucidate a range of perspectives, considerations, and potential implications of hypothetical LGBTQ+ focused studies and results within a large-scale gene-environment interaction research program, the setting in which genomic research regarding sexual orientation and gender identity frequently occurs (Ganna et al. 2019; Hu et al. 2021a, b; Sanders et al. 2021; Zietsch et al. 2021). Here, we report novel findings regarding interviewees’ views of the overall risks and benefits, their personal hopes and concerns surrounding genomic research on sexual orientation and/or gender identity, how genomic research might affect LGBTQ+ populations, and the range of factors affecting the acceptability of such research.

## Methods

This study leveraged qualitative research methods to better understand the deep nuances and complex perspectives of genomic research on sexual orientation and gender identity among a sample of LGBTQ+ participants. We conducted in-depth interviews with individuals (n = 31), recruited initially via LGBTQ+ organizations in the greater Nashville, Tennessee, area, supplemented by snowball sampling of individuals across the country. Eligible participants were aged ≥ 18 years old and self-identified as LGBTQ+, all of whom received the study information found in Appendix A1. Almost one quarter of our sample was recruited from outside middle Tennessee.

Individual interviews were conducted in English via phone in April–June 2020. The interviewers (CHA, CD) facilitated a discussion eliciting participants’ perceptions and opinions on genomic research using SOGI data from LGBTQ+ individuals. Interviewers used a semi-structured interview guide developed after conducting a comprehensive literature review and with feedback from the Program for LGBTQ Health at Vanderbilt University Medical Center. The instrument was pilot tested and revised in iterative rounds to ensure validity. (See Appendix A2 for the interview guide). The final instrument utilized standard open-ended question stems and probes in three distinct domains/sections: a hypothetical large-scale gene-environment interaction research study (i.e., a precision medicine research project called “the National Research Project” [NRP]), and two hypothetical research uses of SOGI data from the NRP: genomic research focusing on health conditions that more often or more seriously affect LGBTQ+ individuals, and genomic research focusing on identifying genetic contributions to sexual orientation and gender identity themselves. Here, we present key findings relating to genomic research using SOGI, electronic health records, and other biobank data to investigate genetic contributions to sexual orientation and gender identity.

Each interview began by asking interviewees to describe their sexual orientation and gender identity. Interviewers then presented a brief educational component of basic concepts in genetics, focusing on essential information to establish a basic understanding of the topic, scrupulously avoided using terms suggesting genetic determinism, such as “gene(s) for,” followed by a description of the NRP and an opportunity for questions and clarifications (the semi-structured interview guide can be found in Appendix A2). Opinions about positive and negative effects were elicited using open-ended prompts. The purpose of these components was to establish a foundation for eliciting informed perspectives and opinions, rather than inadvertently documenting misunderstandings. All interviews were audio recorded and professionally transcribed. Interviews lasted an average of 45 min and participants received $75 for their time. The Vanderbilt University Institutional Review Board deemed this research exempt under 45 C.F.R. §§46.104(d)(2)(ii).

We used an applied thematic approach to code, analyze, and interpret qualitative data. Each transcript was reviewed by at least two members of our interdisciplinary research team (see Appendix A3 for the Consolidated Criteria for Reporting Qualitative Studies [COREQ]), including at least one self-identifying LGBTQ+ team member who independently identified potential codes capturing emergent thematic elements. Six team members (CHA, EWC, LS, KG, AL, AE) then compiled, reviewed, and consolidated codes to construct an initial codebook; two coders (CHA, CD) independently applied the codebook to one transcript, then met to compare code applications, resolve disagreements, and revise the codebook. This iterative process continued until the coders reached ≥ 80% agreement on all code applications. One coder (CHA) then independently coded all transcripts; all coding was reviewed by at least one other team member for accuracy and completeness. Additional methodological information is set forth in the COREQ provided in Appendix A3.

We have included the approximate proportions of interviewees who directly addressed various domains to indicate how many people volunteered particular themes in response to purposely broad, open-ended questions in a semi-structured interview study. Estimates should not be interpreted as a definitive quantification of qualitative data. For example, a percentage of participants addressing any given theme does not necessarily reflect the salience of a theme within or among interviewees, nor does it accurately reflect its perceived importance or potential impact.

## Results

### Participant characteristics

We interviewed 31 individuals representing a range of perspectives and demographic diversity (see Table 1: Self-Described Participant Demographics). More than half of the sample identified as gay or lesbian; 19% identified as bisexual; and 26% identified as other sexual orientations. Nearly two-thirds of our study participants identified as cisgender; approximately 13% and 19% identified as transgender or nonbinary, respectively. Eighty-one percent of the sample identified as White, 16% identified as Black, and 6% identified as Hispanic. Most participants were younger and between the ages 18–39 years (65%), and most participants had a bachelor’s degree or higher (74%), although less than 20% had substantial expertise in science.

**Table 1:** Self-described participant demographics

	n	%
Sexuality		
Gay or lesbian	17	55
Bisexual	06	19
Pansexual	02	06
Queer	05	16
Uncertain	01	03
Gender		
Cisgender	20	65
Transgender or of trans experience	04	13
Non-binary, gender non-conforming, gender fluid, or other gender not otherwise captured	06	19
Race		
Asian	01	03
Black, African American	05	16
White	25	81
Ethnicity		
Hispanic, Latinx, or of other Spanish origin	02	06
Not Hispanic, Latinx, or of other Spanish origin	29	94
Age (in years)		
18–29	12	39
30–39	08	26
40–49	04	13
50–59	02	06
≥ 60	01	03
Not reported	04	13
Education		
High school, some college	06	19
Associate’s/vocational degree	02	06
Bachelor’s degree	14	45
Graduate or professional degree	09	29

### Key context and framing

Participants identified and discussed a range of potential benefits, hopes, risks, and concerns relating to genomic research using SOGI data and the role of genetic contributions to sexual orientation and gender identity from the perspectives of what it would mean for themselves and their communities. Notably, a large majority (~ 75%) of participants assumed that this research would discover some genetic contribution to sexual orientation and/or gender identity, with only three interviewees explicitly presuming no genetic contribution exists and is thus not discoverable through such research. As a result, we specifically asked interviewees to discuss the potential for such research to undermine or challenge evidence of genetic contributions to sexual orientation and/or gender identity to better understand their conceptualizations and latent perspectives regarding the meaningfulness of genetic contributions to their sexual orientation and/or gender identity. In other words, regardless of its potential representation of reality, a hypothetical scenario was used to explore deeper nuances by asking participants to imagine a context contradictory to their established beliefs.

### Perspectives on genomic research on sexual orientation and gender identity

Overall, nearly all participants anticipated that the results of genomic research investigating the role of genetics in sexual orientation and/or gender identity would substantially affect LGBTQ+ individuals and communities and identified a wide spectrum of potential benefits, risks, and harms from personal and community perspectives Participants views were informed in many cases by their perspectives about the unique socio-cultural and familial contexts of LGBTQ+ people and the role of societal systems, structures, and experiences in developing and understanding their individual identities—specifically, their own self-perception, personhood, expression, relation to others, and way of being in the world. Thus, particularly salient contextual themes included common experiences relating to stigmatization and mistreatment of the LGBTQ+ community, including “*anxiety, isolation, and trauma-related incidents*” (P02), “*discrimination or stigma or violence*” (P14), and “*bigotry*” (P09), often within their own families, religious groups, employment, neighborhoods, and other social contexts.

#### Value and informativity

When discussing the potential value and impact of such research, the majority of participants expected that—regardless of whether it confirmed or disputed evidence of a genetic contribution to sexual orientation and gender identity—the new knowledge gained would provide “*more insight, more understanding*” (P08) to “*teach us why we are the way we are*” (P24), anticipating “*conclusive and factual*” (P02) information about their identities, experiences, and communities. Half of participants (~ 50%) described this as potentially beneficial, citing existing health disparities and other inequities resulting from the underrepresentation of LGBTQ+ populations in research. They hoped that new information would relieve the cognitive, emotional, and social burdens and traumas from ongoing narratives challenging the legitimacy of LGBTQ+ identities and experiences (e.g., “‘*were you born this way or you weren’t born this way?’, ‘Is it a choice or is it not a choice?,’ ‘Is it nature or is it nurture?*’” (P07)).

Others, however, were skeptical that this research would be informative. They (~ 40%) emphasized potential challenges unique to LGBTQ+ identities and experiences that could undermine the validity of genomic research on sexual orientation and gender identity; specifically, they doubted the usefulness of SOGI genetic data overall because “*people within our community define* [sexual orientation and gender identity] *different ways*” (P29) and “*those things can mean a zillion different things to different people*” (P16), and described sexuality and gender identity as being fluid, nuanced, or changing over time (*i.e.,* a social construct).

About half (~ 50%) of the interviewees described research investigating genetic contributions to sexual orientation and/or gender identity as inherently cis/heteronormative, designed to benefit non-LGBTQ+ people rather than actually addressing LGBTQ+ identities and experiences:

Research like [this] happens because there is an unwritten heteronormative, cisgender normative script that society has created. And when people go off that script, the people who *are* following the script try to figure out why are other people deviating from it, to relieve their own confusion about something that they shouldn’t even be confused about in the first place. It's trying to pin it on something that, ‘if this is something that people can’t help and is no fault of their own, then maybe we shouldn't be discriminatory,’ rather than taking [LGBTQ+] people at what they say and what is true. It’s research that in itself is based in bias and misunderstanding and just not believing people in the community. So, I think it would be harmful, a waste, and it really just speaks to the undertones that *really* it’s more about [non-LGBTQ+] people being uncomfortable with something. (P17)

Many (~ 40%) interviewees were wary of research that may affirm LGBTQ+ identities within already marginalized sociocultural contexts: “*what it’s affirming is the approval of a system that excluded them in the first place*” (P17). A few (~ 15%) went further, explaining that because they do not feel bound or restricted to the social constructs of sexual orientation and gender identity in the first place, the approval or validation of the social system ascribing meaning to sexuality and gender identity is irrelevant—or even harmful—to them:

Basically, everything is socially constructed; we construct these ideas of what gender is and what sexuality is and what marriage is and what education is, and even what time is. So, we have all these constructions that we believe to be fixed, but […] it was people that invented it in the first place, [and] it takes people to buy into it, to maintain it. So, for me, it wouldn't make a difference because I have an understanding that it's all constructed [and] I'm rejecting the script anyway. (P17)

Overall, about half (~ 55%) of our interviewees opined that research investigating the genetic contribution to sexual orientation and/or gender identity would be relatively low priority, potentially wasting limited resources (*e.g.*, funding, time) that would be better used for research on critical LGBTQ+ issues, particularly research focusing on LGBTQ+ health:

There are too many other ways we can spend research dollars that are more important than figuring out why I'm queer. If one day we have unlimited resources, then sure […] but I don't see it as being a priority for research. (P13)

#### Genomics and agency

Throughout these interviews, one of the most salient themes raised by participants was the role of “choice” in sexual orientation and gender identity. Many (~ 40%) were confident that definitive evidence of genetic contributions to sexual orientation and/or gender identity would *improve* public, medical, and social understanding and perceptions of LGBTQ+ identities and experiences:

It’s increasingly clear that we need to really help the public understand the importance of knowing that sexual orientation is not a choice. I think having the genetic data that, hopefully, backs that up is increasingly encouraging for just promoting overall public understanding and acceptance of the LGBT community. And we've made great strides in the past few decades alone, but I think it's still very much a work in progress. (P10)…

while others were more skeptical:

I think that would probably allow for a doubling down on the whole 2000s era rhetoric of ‘it's a choice.’ […] I can see how it might be validating to some [LGBTQ+] people to feel like, ‘I have this proof that that argument is clearly wrong.’ But it won't stop people from constantly repeating it. […] Some people might find it individually validating for a while, but I'm deeply concerned about what's going to come after. […] When you have a bunch of people whose intuition is ‘ugh, trans—icky’ or ‘ugh, gay—icky,’ it doesn't matter what evidence you put in front of that person, they're just going to alter their argument to fit their intuition. (P31)

Yet, about one-third of interviewees highlighted the concept of choice as more empowering and validating than genetics, positing that narratives focused on genetics can undermine respect for and value of LGBTQ+ persons’ personhood, free will, and agency:

When I accepted myself and was fine with having sex with or being in a relationship with people of the same gender, I found it rewarding. I found that I was still healthy and sane and able to live my life. I think it turns on me to ask, ‘…do I *know* that I’m gay? Will this change at some point?’ […] maybe I *don’t* fully know myself, and maybe in 10 years I'm going to feel like a different person – and that’s okay! Now that I've already come through a lot of that journey, it would be just more acceptance of myself and open to exploration, whereas if I get a confirmation that there is a genetic cause, then I’m not going to be questioning much anymore. (P22)

Even if I chose this, why would me choosing to be with someone of the same gender, why would me choosing to be what gender I am – why would that matter? There was this whole campaign, ‘it's not a choice’ and ‘I'm born this way,’ that type of mentality. But that whole idea just goes to the unwritten script of heteronormativity and cisgenderness: ‘why would you choose to go against it [since] it is something that’s desirable and normal? So if you step out of it, there has to be some validated reason—like your genetics—that deviate you from that.’ (P17)

Half (~ 50%) of our interviewees anticipated that research undermining or challenging evidence of genetic contribution to sexual orientation and/or gender identity would perpetuate and “*fuel the flames of the argument that being* [LGBTQ+] *is a choice*” (P21), which may exacerbate self-doubt, emotional distress, and cognitive dissonance within LGBTQ+ individuals and communities.

About one-third of the participants indicated that genetics is—or should be—immaterial to LGBTQ+ identities and experiences:

We shouldn't be concerned about the biological basis of gender identity and sexual orientation. There might be some scientific medical benefit to it, but ultimately, we shouldn't be concerned with why people are queer at all – we should be concerned with the fact that people *are*. I don't care *why* someone is gay or trans or non-binary; there are people who are in these categories, how can we help them out in the day-to-day?’. What will help them is saying that it doesn't actually matter *why* you're in these categories or not, it just matters that you *are*. (P31)

#### Vindication and validation

Almost all (~ 75%) interviewees spontaneously discussed the pros and cons of this research assuming that it would confirm their personal beliefs that genetics does influence sexual orientation and/or gender identity. That is, nearly all participants assumed that results from such research would confirm their existing self-perceptions:

It would just help confirm something that I already know. I already know that my sexuality is not a choice. I know it's a part of my genetic makeup and what makes me who I am. (P06)

Half (~ 50%) of participants expected that research *confirming* a genetic contribution to sexual orientation and/or gender identity would provide external validation of LGBTQ+ identities and experiences, contradicting public misperceptions of LGBTQ+ identities and experiences. Many participants hoped that people outside the community “*would start to come around more in the fact that people are who they are*” (P08) and that LGBTQ+ identities and experiences would be more respected and valued on societal and personal levels:

If genetics are involved, it gives the sense of, to me, almost automatic validity to someone's [sexuality or gender]. And people have been having to prove themselves to how their life is lived out, that they are valid, or their relationships are of value. […] To see how genetics play a role or not and if there is some sort of relationship between genetics and sexual orientation or gender identity, then it would be great for that to be something that's standard in education or standard understanding in the medical community, even more so than it is. I think there could be an opportunity for more education, which would hopefully lead to more societal acceptance of LGBT persons. (P22)

Half (~ 50%) of participants anticipated feeling vindicated and hoped that their identities, experiences, and relationships would be further legitimized, respected, and valued, particularly within their personal families and communities (*e.g.*, religious groups, employers):

I would maybe feel validated—for my family, who for a long time wanted me to be straight, and I, who wanted myself to be straight for a long time and went through a lot of just trauma to try to change my sexual orientation. It would be maybe a little bit validating to be like, ‘Look, it's in my genetic code. It can't be changed.’ But I already know that. I already know that it can't be changed. (P15)

Many (~ 30%) interviewees anticipated that research confirming a genetic contribution to sexual orientation and/or gender identity would also increase internal validation, acceptance, and self-understanding within LGBTQ+ individuals themselves. Yet, nearly half of our interviewees (~ 45%) said findings of genetic contributions would have little-to-no effect on their personal identity or self-perception *now* but compared the impact of validation against their *former* selves, and emphasized its importance to current and future LGBTQ+ youth:

As a person in this stage of life that I am now, I don't know that I have that many worries or misgivings [about genomic research on sexuality and/or gender]. But I'm an independent adult and have social stability and economic privilege. So, those things certainly play into me having less anxiety initially about thinking and processing that sort of potential for myself. (P28) Would that make me feel better about who I am now? No. But it might have back in the early days of my coming out of the closet. . . .I feel very secure in who I am. I feel affirmed in who I am. I have several resources that I can go to that would validate me the way I am now. Back then, I didn't have any of that. I had no role models, no books, no anything. I was flying by the seat of my pants wondering what the hell was going on. (P20)

A lot of people who identify as LGBTQ, or who perhaps haven't fully come to terms with their identity, could potentially really benefit from something like this to sort of shift that blame away from ‘this is something that I caused.’ I come from a pretty conservative background and if this type of research had been available when I was 12 and trying to figure out what my sexual identity was, I probably would have taken some comfort in knowing that it was a real feeling and it wasn't something that I could necessarily change. […] When I was 12, 13, 14, I actually remember searching ‘gay gene’ on Google and really trying to understand was this something that was beyond my control: was there a reason for why I was born this way? That would give me a lot of strength. I think for others it might do the opposite, but for me personally I know that it would [have given] me a lot of strength. (P22)

#### Invalidation

A little more than half (~ 55%) of our participants stated that research confirming a genetic contribution to sexual orientation and/or gender identity could invalidate LGBTQ+ experiences and identities:

It kind of dismisses people's own agency in being able to lay out who they are and have that time and space to figure out who they are. (P05) It would kind of invalidate the journey that I've been through so far because I've identified differently throughout my life. Even though I've found a label and identity that I feel very comfortable with now and that suits me well, it would make me feel weird that there was any genetic influence in my sexual orientation. (P21)

These participants explained that because they spent decades expending a lot of cognitive and emotional energy and often endured substantial psychological, social, and familial trauma in understanding their sexual orientation and/or gender identity*,* it could be distressing, disappointing, or even confusing to learn that an identity that is fundamental to their personhood is, at least to some extent, influenced by their genetic makeup:

My identity and who I am is a lot of hard work and time and processing on my own part. I’d be like, ‘am I just the result of a random mutation in genetic copy? Is that really what this huge part of my identity comes down to?’ I think that would be a little bit sad for me. (P15)

#### Inciting division

About a third worried that research confirming a genetic contribution to sexual orientation and/or gender identity could exacerbate these harms *within* LGBTQ+ communities:

I think that that would then introduce another hierarchy within the queer community, like, ‘I have this thing that says I *actually am* gay, or I *actually am* trans.’ And there will be an underclass of people that would not be able to get that information because they don't have access to medical care. And that would necessarily impose more poverty and stratification within our own community. (P29) It has this really bizarre way of identifying or having this threshold that's like, ‘You're only gay if you have this certain genetic makeup […] You don't have this makeup, so you're not really “pure” in that way,’ which just really grosses me out to think about. (P27)

Overall, while most discussed the effects of personally carrying a genetic variant influencing their sexual orientation and/or gender identity, over half worried about the implications of *not* carrying it:

As somebody who's lost everything to come out and be gay, I would be terrified that they would find a common gene and I *didn't* have it. And that I would feel like less of a gay person. […] Did you ever read the book ‘Sneetches on the Beaches’? There are these creatures and they're identical except that some of them have these stars on their bellies. And eventually those Sneetches become the societal leaders, they're the popular ones. Everybody wants to have a star. It almost sounds like a star-bellied Sneetch situation—am a second-class queer if I *don't* have the genetic mutation? (P09)

#### Pathologization and medicalization

Nearly half (~ 45%) of participants were wary that research messaged as investigating a “genetic variation” related to marginalized sexualities and/or gender identities would further stigmatize people who are LGBTQ+:

Saying ‘a genetic variation’—a ‘variation’ means it's not normal, or atypical. If you're finding a variation of a minoritized group of people, then it seems like a deviance or something that shouldn't have happened or is happening in a much [less frequent] format. And so, you're creating something that is othered. You're othering a group of people now that has been othered forever in a culture lens, but you're now doing it in a scientific, health, and research way. (P27)

These interviewees anticipated that research confirming a genetic contribution to sexual orientation and/or gender identity would lead to medicalization of LGBTQ+ identities and experiences:

I would really, really be concerned about pathologizing any sort of LGBTQ identity. […] There are folks who would feel really traumatized by knowing that something is inherently ‘wrong’ with them […] I would be scared that it might become super medicalized and it might be perceived as an illness. And then I would worry, would they—just like how [people] might rally around someone who has cancer—support them in ‘overcoming’ or ‘battling’ their ‘illness’? (P12).

Respondents also discussed that discovering associated genetic variations could affect health and medical care. Some (~ 30%) hoped that research confirming a genetic contribution to sexual orientation and/or gender could increase access to health care and other resources “*to support* [LGBTQ+] *people and parents better*” (P26), particularly for transgender youth:

It could be helpful for young kids trying to make some pretty significant health decisions and their parents are just trying to do what's best for them, and they're trying to understand all this. When you're on a certain timeline, before [trans] kids hit puberty, maybe that could be helpful to be able to learn. It could make it easier for kids to get the health care they need. (P30) [This research could] help younger trans folks transition safely. Because a lot of the debate when parents have a trans teen is, ‘You can't let them transition. They're too young. It might not be safe,’ or whatever. But then how much benefit comes from trans kids being able to transition before they hit puberty? So, [this could] help them get the results that they need in a way that's still safe for their growing bodies and things like that. (P26)

However, other interviewees (~ 15%) were concerned that such research would exacerbate barriers to health care and increase medical gatekeeping**;** one trans participant described her personal experience in echoing this concern that “*in order to get the medical care that someone desires, they would need to take a blood test to determine whether or not they fit the criteria of somebody who is* [LGBTQ+]” (P29):

There are beliefs, especially within the medical community already, that you have to have gender dysphoria in order to be considered a trans person and medically transition. […] If that is the standpoint that you're starting from, of ‘how do we figure out if this person is trans?’ and just believing [that] you look for something like gender dysphoria, I'm telling you that that is way off. For somebody like me who is pursuing vaginoplasty and things like that, I have to get three permission slips from three people who say ‘yes, this person has a long experience with gender dysphoria dating back from when she was younger.’ And to tell you the truth, I would say that's not even true; I have to just parrot these things, and I know other women who have to parrot these things in order to get the things that they need. (P29)

#### Genetic Editing and Erasure

Almost half (~ 45%) of the participants feared the “weeding out,” “genetic editing,” or erasure of LGBTQ+ individuals. Specifically, these interviewees anticipated threats to reproductive rights of LGBTQ+ individuals:

I, a lesbian, gave birth to a child that is genetically mine and is trans. Does my ‘gay gene’ contribute to having some potential ‘trans gene,’ as well? People that are fighting against gay rights—would that make them more inclined to say, ‘You're going to create more of the gay babies. We're not going to let you have these.’? (P09)

as well as negative implications in reproductive decisions of non- LGBTQ+ people:

As IVF and genetic screening and genetic counseling continue to progress, I'd really be worried about couples coming in and screening out embryos who have a propensity or who might turn out to be LGBTQ. I think that would be so borderline eugenic and so counter to what we are trying to do for LGBTQ visibility and equality. (P12)

A quarter of our interviewees explicitly referred to a threat of “*cleansing or eugenics*” (P10).

Since the LGBT community is considered undesirable members of society by a good enough chunk of society still, there’s a danger of and potential for eugenics-like movements to be had if it were found that there was a genetic factor in being [LGBTQ+]. (P21)

The main thing I would be concerned of is an attempt by some parties to ‘wipe out’ the community, try to either prevent fetuses with those genes from being born or trying to come up with genetic treatments to eliminate that genetic sequence that results in being gay or trans—trying to find a cure for being LGBTQ, essentially. (P21)

#### Concerns about social harms

Regardless of such research’s conclusions regarding the genetics of sexual orientation and/or gender identity, many interviewees (~ 30%) foresaw an increased risk of violence against LGBTQ+ individuals and communities, with regular references to escalating discrimination, stigmatization, and other abuses such as “*reparative therapy*” (P02). Interviewees (~ 40%) reflected broad concerns about intentional and unintentional misrepresentation or misuse of data or findings. Overall, these participants generally were not concerned about reckless or malevolent actions by researchers, but fully anticipated that ethically appropriate and scientifically legitimate research—regardless of whether it confirmed or undermined evidence of a genetic contribution to sexual orientation and/or gender identity —would be weaponized by others. Many (~ 30%) interviewees were wary that research findings could be used to attack LGBTQ+ people’s rights. These interviewees were leery of centralizing data from vulnerable individuals—particularly given potential access by government, law enforcement, and unknown researchers and entities—and were wary of the ability to find, track, and “*corral folks*” (P18) who are LGBTQ+:

Let's assume for a second that this study [confirms] a genetic marker for transness, hypothetically. Then the government decides that they want to round up trans people and put them in camps. […] I don't have a lot of faith in the government to maintain protection for LGBTQ people, particularly when it comes to trans rights right now. (P31)

Others (~ 30%) anticipated discriminatory uses of research results undermining evidence of a genetic contribution:

That’d bring some detriment to gay rights. There would be folks who would say, ‘you choose to be this way, therefore you shouldn't have rights.’ (P25)

Concerns of weaponization were particularly salient in discussions of research results identifying a genetic variant contributing to both sexual orientation and/or gender identity as well as a health condition:

If the study comes out like, ‘being queer is genetically related to poor mental health,’ that would be disastrous. Imagine trying to — *having* to — defend against that. That's a whole other vector of psychological attack on the queer community. And that's already something that we’re seeing; you'll find Neo-Nazi fasc[ist] types online posting about trans suicidal rates constantly. Imagine if they had even more ammo on that; that's horrific. You might end up [finding that] there's this gene and then figure out a way to use that to improve [health]. That might be good, but you would still have to deal with that psychological socio-cultural fight over the discovery of the ‘queer gene,’ which would be such a nightmare. Let me put it this way: anything negative that is correlated with those studies would have really horrific social implications. I don't know if we're there. I don't know if we would ever get there, but we're sure as hell not there right now. (P31)

### Acceptability of Genomic Research Investigating Genetic Contributions to Sexual Orientation  and/or Gender Identity

After considering the range of potential risks and benefits, respondents were asked to indicate a binary (yes/no) opinion of whether genomic research investigating the genetic contributions to sexual orientation and/or gender identity is acceptable. About half of interviewees said such research is acceptable (n = 14), primarily prioritizing the potential social and medical value and other benefits for other LGBTQ+ people now and in the future:

I think it's important, and I would participate. […] But whatever the result was — whether yes, there's a gene or no, there isn't a gene—it's not going to change who I am. But I think it's important globally, and for generations to come, it would be an answer to an age-old question […] It's not going to change [me] personally, but it's going to have an impact on broader society. (P25)

Others (n = 18) disagreed, opining that such research is not acceptable. These interviewees emphasized the aforementioned risks and potential harms which they perceived to outweigh potential scientific or social value:

I cannot ponder a situation right now where I think that would be an acceptable type of research. […] I don't know why we would need to know that. And I think even if I was convinced that there was a reason, the implications for negative use of that would just be so potentially wide ranging, that I don't think I would want that sort of research to exist. (P28)

It's playing with fire. It's playing with lit matches in a pool of gasoline. […] Anything neutral or positive might have some good outcomes, but it would have a lot of negative outcomes associated with it. So, you might end up saying, ‘we found this gene associated with this characteristic’ and then figure out a way we can use that to improve [a health condition]. That might be good, but you would still have to deal with that psychological social-cultural fight over the discovery of ‘the queer gene,’ which would be such a nightmare. I don't know if we would ever get there, but we're sure as hell not there right now. (P31)

### Strategies to improve research and its acceptability

Regardless of their response regarding acceptability, interviewees were prompted to generate a range of measures that could increase the acceptability of genomic research using SOGI data to investigate genetic contributions to sexual orientation, gender identity, and LGBTQ+ health. Many (~ 30%) interviewees emphasized the fundamental importance of researchers’ respecting LGBTQ+ experiences and identities as legitimate, valid, and valuable beyond the research context:

Listen to them. Just listen to them and their stories. While that may not be genetic data coming out of them, it's their heart. (P01)

These interviewees also highlighted the necessity that researchers develop a meaningful appreciation of the context in which LGBTQ+ -focused research is conducted, “*the responsibility that they hold by holding this database*” (P03), and potential risks and burdens to LGBTQ+ individuals and communities:

Science is not black and white. It's affected by culture and society, and it affects culture and society. So, think about why you want to do certain research, and also think about how it could affect the communities at hand. Because the need for knowledge to exist is not more important than the people that it could possibly harm. (P03)

I think that the important thing would be to tell them how to engage being aware of the systemic oppression that they're either recreating and perpetuating, or undermining, through their study. (P16)

Just think of every possible ramification of every possible version of your findings that there are. Because while you're doing this with good intentions, your results could be used by any people in any way, so just be cognizant of that. (P21)

Several (20%) called for assurance that research would be designed to benefit LGBTQ+ people:

It is paramount and critical for any kind of research that's done on our community be done with the sole purpose and intent of moving the community forward to live fuller, happier, richer, thriving lives. […] Is the research going to help us understand something that is affecting our community in some way, shape, or form? The result must be that it's going to aid our community and move us forward to experience more success and have an opportunity for greater opportunity. (P02)

Many (~ 30%) interviewees emphasized privacy and confidentiality considerations unique to LGBTQ+ individuals and other marginalized and stigmatized groups, calling for additional and/or reinforced protections both within and beyond the research design:

It's really about recognizing that there's always going to be risk with identification when you open yourself vulnerably. You want to make sure that that information is safeguarded and trusted. […] The biggest risk is really this forced outing and re-traumatization that I think many people might experience. […] I think that it would be incumbent on [researchers] to think about the wording of certain questions and to think about how LGBTQ people are already sometimes inherently distrustful of the medical establishment. So, recognizing that by asking them to be this vulnerable and give you their DNA, the core essence of who they are, you really have to take that [seriously] and you really have to accommodate their unique societal and emotional needs. (P12)

A specific strategy suggested by many participants (~ 25%) was “*interacting directly with LGBTQ people*” (P12) in meaningful engagement with LGBTQ+ communities, with “*not just a token move at bringing in LGBTQ voices*” but, instead, “*continual widespread soliciting of feedback and incorporation of LGBTQ voices*” that is “*ingrained in the* [research] *process*” (P28):

Even if the researchers themselves are [LGBTQ+], make sure to consult with other people because they're just one person; experiences aren't universal. (P21)

Several participants (~ 15%) proposed that, ideally, genomic research using SOGI data should be conducted by investigators who themselves identify as LGBTQ+, but most stopped short of calling it necessary to conducting ethically appropriate research on sexual orientation, gender, and/or LGBTQ+ health:

Of course, I would want to say, ‘I think they should all be queer,’ but then I also think about if I have a heart issue, I'm going to the top heart surgeon or doctor in the nation, I don't care if they're gay or not — I want them to do the best work they can. [But] in my daily life, I dedicate to working with people in the community and supporting queer businesses and queer doctors, so I would feel more comfortable if they were queer in a broad sense. (P27)

A few (~ 10%) participants went further, opining that LGBTQ+ researchers are not only important, but necessary to conducting ethically appropriate research on sexual orientation, gender identity, and/or LGBTQ+ health:

In this day and age, we’ve got enough people who can do this who *are* [LGBTQ+]. Historically, research has been biased [against] us. And other people had [dismissed us] because this isn't their life experience and they're coming at it from a career standpoint and not from a personal standpoint. It's 2020, we got options now. So, let's be intentional about who is in charge of this kind of research. I think it should be us. (P19)

Although, a few emphasized that LGBTQ+ representation among researchers is not in and of itself sufficient:

The oversight needs to be guided by the people who are affected and needs to be incredibly transparent to the population at large. And not just other queer researchers, but regular working-class people who this will primarily affect. Because I would bet that a researcher who makes a significantly more amount of money than, say, some trans person who works at Starbucks so they can actually get insurance to pay for their hormones—they have a different experience. (P29)

Finally, two-thirds focused on the importance of giving additional consideration in several practical and operational areas, such as transparency in consent, specific consent for SOGI data in genomic research, control over third-party access and data sharing, methodology, including “*drawing from a representative sample of the* [LGBTQ+] *population*” (P23); analysis and interpretation, with specific attention to “*intersectionality*” (P26), and dissemination efforts, calling for assurance that “*the public has access to* [a] *layman's term version of what this information means*” because of the “*broad social implications*” (P19) of this research.

## Discussion

Our study participants highlight how learning about genetic contributions to sexual orientation and/or gender identity— particularly in the current social, cultural, and political environment—could have both potential positive and negative impacts on LGBTQ+ individuals and communities, echoing the sentiments and ideas recently reported by Thomas et al. (2020) and Rajkovic et al. (2021). Our findings, however, go further than exploring LGBTQ+ individuals’ views about the acceptability of this research by revealing LGBTQ+ individuals’ nuanced perceptions of the potential power of genetic contributions’ to sexual orientation and gender identity to cultivate or complicate their own self-understanding as well as their relationships with others within and outside the communities with whom they identify.

In these interviews, participants, who for the most part were not experts in science, generally assumed that genetic variation contributes to sexual orientation and/or gender identity, a finding consistent with previous studies (Joslyn and Haider‐Markel 2016; O'Riordan 2012). This attribution is hardly surprising given the iconic place that genetics has held for decades in popular discourse (Nelkin and Lindee 1995; Keller 2000) and the persistence of notions of genetic essentialism, which assert that genetic variation is the dominant factor responsible for human characteristics (Brodwin 2002; Dar-Nimrod et al. 2021; Heine et al. 2017). So-called “born this way” campaigns, which draw on essentialist views of sexuality and gender, declare an innateness and immutability to LGBTQ+ identities and experiences. The phrase, which participants frequently recited and referenced, has been utilized as a rhetorical strategy by mainstream activists to advocate for LGBTQ+ equality (Schilt 2015; Tygart 2000) and support for LGBTQ+ youth (Stein 2007). But while this messaging has had some success (Haider-Markel and Joslyn 2008; Hewitt and Moore 2002; Overby 2014; Wood and Bartkowski 2004), it has also been met with pushback from LGBTQ+ individuals and scholars (Bennet 2014; Schilt 2015).

This history of genetic essentialism and rhetorical strategy may help to explain why many interviewees spontaneously used the term “gay gene” even though the interviewers took care to talk about genetic contribution*s* to sexual orientation and gender identity, rigorously avoiding any suggestion of a major role for single genes. The term “gay gene” entered the public lexicon in the 1990s, where it has remained despite reservations about its validity, (Akpan 2019) its impact on lesbian, gay, bisexual, transgender, queer, and questioning (LGBTQ+) individuals and communities, (Conrad and Markens 2001; Kitzinger 2006; O'Riordan 2012) as well as current scientific hypotheses about the complex contributions of genetic variation to spectrums of human sexual orientation and gender identity (Polderman et al. 2018). The ongoing salience and power of this concept must be taken into account even as efforts are made to move toward more scientifically informed views.

The prospect of defining genetic contributions to sexual orientation and/or gender identity was endorsed by many of our interviewees. Notably, most interviewees perceived this research to be beneficial primarily for non-LGBTQ+ populations. They also reported some potential benefit to interviewees themselves, perhaps increasing tolerance, acceptance, or even affirmation from family, religious groups, government leaders, neighbors, employers, and others, findings consistent with previous survey data showing that genetic attributions for sexual orientation and gender identity may decrease negative attitudes and increase acceptance and support of LGBTQ+ people (Garretson and Suhay 2016; Haider-Markel and Joslyn 2008; Joslyn and Haider‐Markel 2016; Rajkovic et al. 2021; Thomas et al. 2020).

Of particular note, while most of our participants described their present selves as generally secure in their sexual orientation and/or gender identity, many reflected on their personal experiences of “coming out” or living as an LGBTQ+ person, citing the possible utility of understanding genetic contributions to SOGI for navigating a cis/heteronormative world (Morandini et al. 2017). Several specifically emphasized the potential benefits of such research for young people who were exploring their sexual orientation and gender identity, which led some to see research participation as a way of “paying it forward”.

Yet, when discussing genomic research on sexual orientation and/or gender, numerous concerns and reservations arose. Our participants anticipated a range of risks and harms regardless of whether scientific investigations confirmed or failed to find genetic contributions to sexual orientation and/or gender identity. They echoed previously reported concerns about misinterpretation, misrepresentation, and misuse of such research, and they emphasized the threat of pathologizing LGBTQ+ experiences and identities regardless of what the research reveals (Ansara 2016; Rajkovic et al. 2021; Rich 1980; Thomas et al. 2020). Some worried that genomic research using SOGI data would not yield valid results, citing methodological challenges such as the fluidity of LGBTQ+ identities and experiences over time and the range of diverse, often evolving, definitions relating to sexual orientation and gender identity. (Diamond 2021).

Since most interviewees presumed that research would confirm genetic contributions to sexual orientation and gender identity, common apprehensions centered on medicalizing, preventing, or “fixing” LGBTQ+ identities and experiences, including threats to LGBTQ+ individuals’ reproductive rights to prevent or deter procreation, as well as gene editing of LGBTQ+ adults and children. Other common concerns related to issues within LGBTQ+ communities include using genetic results as a basis for gatekeeping of health services or sustaining social hierarchies, personal implications for LGBTQ+ individuals who may not have such genetic variant(s), and surreptitious genetic testing with malintent, particularly in children.

Yet, interviewees were also optimistic that research confirming genetic contributions to sexual orientation and gender identity could improve LGBTQ+ wellbeing by legitimizing and normalizing LGBTQ+ identities and experiences, particularly in familial, employment, and other socio-political contexts. They opined that facilitating access to, and increasing the quality of, health care and other resources for LGBTQ+ individuals (particularly for transgender youth and other marginalized genders) could also be improved with understanding of genetic contributions. Finally, understanding the genetic contributions of sexual orientation and/or gender identity may generally improve public knowledge and acceptance of LGBTQ+ populations as well as advance medical education, training, and treatment towards LGBTQ+ health equity.

When discussing the prospect of research results that failed to uncover genetic contributions to sexual orientation and gender identities, interviewees’ primary concerns were invalidation of LGBTQ+ identities and experiences along with delegitimization of already-marginalized individuals and groups, all of which speaks to the power of the genetics narrative as the foundation of “real differences” in current society. Interviewees foresaw increased prevalence of harmful practices (e.g., so-called “conversion therapy”) and exacerbation of existing barriers to health care and other resources (e.g., gender affirming medical care) (American Medical Association and Health Professionals Advancing LGBTQ Equality 2019; Padula and Baker 2017; Wagner et al. 2021).

Like a number of scholars, (Adams et al. 2017; Blair 2016; Vincent 2018) participants often emphasized investigators’ ethical obligations generally to minimize risks and harms not only to their LGBTQ+ participants but also to LGBTQ+ individuals and communities more generally, often noting the complex and evolving socio-political environment. Our study participants overwhelmingly endorsed continued engagement with LGBTQ+ communities throughout all stages of genomic research studies using SOGI data, echoing recommendations from the International Gender Diversity Genomics Consortium and others (Polderman et al. 2018; Rajkovic et al. 2021). We note, however, that much work remains to be done to develop authentic strategies for engagement over time, (Erikainen et al. 2021; Luna Puerta et al. 2020; Milne et al. 2021) particularly in the setting of large datasets collected for unspecified future research (Watson et al. 2022). Some interviewees went further, suggesting involvement of LGBTQ+ investigators as necessary, though not sufficient, for ethical conduct of genomic research on sexual orientation and/or gender identity. These views support the validity of prior recommendations that a human rights framework be adopted to protect the interests of LGBTQ+ people so that “researchers, community leaders, and members of marginalized populations collaboratively develop long-term, equitable partnerships to inform the design, conduct, and dissemination of research programs tailored to improve health equity” (Polderman et al. 2018) at page 105). The participants further emphasized the crucial ethical responsibility that scientists who endeavor in such research have to steward the use of their results into the future.

There were several limitations to this study. Our sample was not intended to be representative of all LGBTQ+ individuals or communities. Rather, the purpose of our exploratory qualitative study was to add additional, more nuanced insights into the landscape of LGBTQ+ perspectives on genomic research and to identify issues for further investigation. With full acknowledgment that LGBTQ+ communities are not univocal and that no individual is expected to represent others, our study was one of the first to interview LGBTQ+ people about efforts to define genetic contributions to sexual orientation and/or gender identity using current genomic technology. Almost all our participants were from the southeastern US, with almost 90% from Tennessee, which in some ways is a strength given the anti-LGBTQ+ sociopolitical dynamics of this part of the country. Since our interviewees were informed of the opportunity to participate in our study through local LGBTQ+ organizations in Nashville or through word of mouth, most (if not all) had some connection to or involvement with social networks and community support systems valuing LGBTQ+ individuals. Further, our participants were willing and able to self-identify as LGBTQ+ and openly discuss their sexual orientation and/or gender identity in an audio recorded phone conversation with a researcher with whom they had not previously interacted. Thus, our sample does not include LGBTQ+ individuals who are not comfortable with or capable of doing so (e.g., those who are not “out” to some extent, are constrained by their environments, or are generally more reserved in discussing personal topics). We also did not interview minors due to ethical and legal challenges at this exploratory stage. Future research to explore how minors who are exploring their sexual orientation and/or gender identity think that genomic research would affect them and their journey could be valuable. Despite our efforts to maximize diversity, most of the participants were white, cisgender, and had some or higher levels of college education. Future research should consider oversampling LGBTQ+ people of color and those from lower socioeconomic backgrounds. Doing so will facilitate subgroup research within the LGBTQ+ community (e.g., LGBQ + ciswomen, GBQ + cismen, trans men, and trans women) and the impact of intersectionality on attitudes among individuals living at the junctions of multiple marginalized identities (e.g., transgender people of color). Finally, interviews occurred amid several key contextual events, including the landmark U.S. Supreme Court decision that Title VII of the Civil Rights Act of 1964 protects LGBTQ+ people from employment discrimination on the basis of their sexual orientation and gender identity (Bostock v. Clayton County, 2020); the initial surge of the COVID-19 pandemic; and the threat of several “anti-transgender” initiatives at federal, state, and local levels, such as bathroom bills and requirements for transgender athletes to participate in sports based on their sex assigned at birth rather than their gender identity (M. Tyler Gillett 2021), which increased dramatically after these interviews were conducted.

In summary, our interviews illuminated myriad complexities, tensions, and nuances related to research regarding genetic contributions to sexual orientation and gender identity as well as LGBTQ+ health. Our study of LGBTQ+ people and their perspectives on genomic research will help inform whether and how best to support, design, develop, describe, conduct, report, and implement genomic research on sexual orientation and gender identity to maximize benefits and minimize harms. This project also underscores the need for further work with people who are LGBTQ+ to understand and address these challenges in greater detail. Particular attention is warranted for marginalized sexualities and genders, access to and quality of health care for LGBTQ+ populations, recognition and validation of LGBTQ+ communities, potential harms, intersectional health needs, and the complex socio-cultural landscape in which LGBTQ+ communities live. Navigating this landscape will surely be challenging, but if done well, may provide the foundation for ethically acceptable genomics research in this domain.

## Data Availability

These interviews often contained information that was highly sensitive that can only partially be protected by de-identification. As a result, the IRB application stated that data would be securely stored and that Dr. Clayton “will maintain the research data and will restrict access to the key study personnel.” We are, however, willing to share these data with investigators who are willing to comply with the same requirements that applied to our team, that is, to sign data use agreements that preclude further data sharing and efforts to re-identify participants and to do research focused only on attitudes of individuals who are LGBTQ+ about genomics research, i.e., the original purpose of the study. Investigators interested in such access may contact Catherine Hammack-Aviran (Catherine.m.hammack@vumc.org) or Ellen Wright Clayton (ellen.clayton@vumc.org).

## Appendix

### Appendix A1. Genetic Privacy and Identity in Sexual and Gender Minorities: GetPrISM Information provided to participants

**INTRODUCTION:** We are asking you to take part in a telephone interview for a study called *Genetic Privacy and Identity in Sexual and Gender Minorities: GetPrISM*, which is being conducted by Vanderbilt University Medical Center. This study is supported by a grant from NHGRI (R21 HG010652-01).

**PURPOSE:** The purpose of this study is to find out what members of the LGBTQIA + community think about genetic research using medical record data, including sexual orientation and gender identity.

**HOW THE STUDY WORKS:** If you are eligible and decide to take part in this study, we will invite you to participate in a one-time telephone interview. The questions we ask will be about your thoughts about genetic research. We will only ask for your opinions; we will not ask personal questions (for example, about your health or genes), and we will not ask you to have genetic testing done. You do not need any special knowledge or experience, and there are no right or wrong answers. The interview will last about one hour, and we will audio record the interview to help us remember your feedback.

**RISKS:** The risks of taking part in this study are very low. We are just interested in learning what you think about genetic testing using sexual orientation and/or gender identity data. Instead of using your name, we will use a code number. A list linking the code to your name will be kept secure so that only the researchers can see it. We will not use your name or other identifiers in any reports or presentations about this study.

**BENEFITS:** There are no direct benefits to you for being in the study. The reason someone might like to participate is to help researchers learn more about how members of the LGBTQIA + community think about genetic research using sexual orientation and gender identity data.

**COSTS/PAYMENT:** If you participate, there are no costs to you, and you will receive a $50.00 gift card for your time.

**ALTERNATIVES:** You do not have participate in this study. If you do participate, you may choose not to answer any question and may leave the discussion at any time.

**CONTACT:** If you would like to participate in this study or if you have any questions, please contact the researchers at Vanderbilt by calling Carolyn Diehl at [redacted]. You may also contact the Vanderbilt Institutional Review Board (IRB) Office at 615-322-2918 (IRB #: 191388).

In addition, prior to the interview, participants were told. We are conducting interviews to hear people’s thoughts about genetic research in the LGBTQ+ community. My role is to listen to what you have to say and report it back to the researchers. There are no right or wrong answers to my questions I will ask---only opinions. Hearing *your* perspective is important to us, so any thoughts, opinions, or experiences you are comfortable sharing with us will be very helpful. If I ask questions that you don’t want to answer, that’s fine; just let me know and we’ll move on. If you decide part way through the interview that you no longer want to participate, just let me know and we can stop. Finally, I’ll ask you to imagine some hypothetical [pretend] research projects. We are not doing any of these types of projects, and we are neither for nor against these types of research.

- Do you have any questions?
                            Yes   No
- *Verbal agreement to participate*
                            Yes   No
- *Verbal agreement to be audio-recorded following disclosure of reason for recording and plan for redaction of identifiable information*
                            Yes   No

### Appendix A2. Interview scenarios and question topics

Introduction

Your health is the result of a very complex interaction among many factors: what is in your genes, or your DNA; where you live, what environments are you in; your lifestyle and health behaviors, like do you exercise, do you smoke, what does your diet look like; etc.

For example: your employment situation, your insurance coverage, your family life, and your social environment, and even stressors in your life like discrimination can all affect your cortisol and blood pressure.

So, knowing your genetic makeup can tell you *something* about the chance that you have or will develop a complex health condition, like high blood pressure, but it can *not* say for sure whether you will.

- Assess understanding; probe for misconceptions
- Opportunity for questions

**1. National Research Project**

**Table:**

The National Research Project (NRP) is a large-scale research study to collect and store many different types of information from more than one million people in the United States. The goal is to facilitate research by creating one central resource of data and specimens for researchers to use for a range of studies well into the future. Researchers hope that by taking into account individual differences in lifestyle, environment, and biology, they can find new ways to predict, detect, manage, and treat disease and improve health for everyone
The NRP is currently inviting adults to participate in this historic research project. Like most precision medicine research studies, the NRP can collect and store the following:
i.Biospecimens (e.g., blood), including genomic information/DNA
ii.Electronic health record (EHR) information from their healthcare providers, such as their current and past diagnoses and medications, family health history, test results, gender identity and sex assigned at birth, and sexual orientation
iii.Participants’ survey responses about their lifestyle and health behaviors, such as smoking, exercise, sexual activity, stress, and environmental exposures
In order for the project to be effective, it is important that the participants broadly reflect the diversity of the United States. For example, researchers want to be sure that people of different races/ethnicities, ages, genders, and other characteristics are included in the study. This is important because unless all types of people take part, researchers can’t be sure that their results will apply/be relevant to all people, including LGBTQ+ people
Individuals who want to participate will give broad consent to research on an array of topics by an array of researchers using their specimens and data in the future
Researchers apply to get access to the specimens and information stored in this Biobank. If approved, they will use de-identified health information and/or biospecimens (blood, saliva, tissue), so they will not know who the data came from. There is a master key connecting code numbers to individuals’ identities, but researchers cannot access it. There are many other safeguards in place to protect the specimens and data from unapproved access and unapproved uses
Researchers can link biospecimens, EHR data, and survey responses to get a wide range of health-related information about a person, even though researchers don’t know the participant’s identity

- Initial reactions to NPR
- Potential benefits of NPR; personal hopes relating to NPR
- Potential risks/harms of NPR; personal concerns relating to NPR
- Likelihood of participating in NPR

**2. Genomic Research on LGBTQ+ Health**

**Table:**

Researchers who want to learn more about **health and disease in ** LGBTQ+ **communities** would use blood samples from the NRP biobank to study the DNA / genetic makeup of participants who identify as LGBTQ+
Researchers can use special computer systems that use EHR and survey data to find which biospecimens and other data belong to NRP participants who are LGBTQ+ , but they wouldn’t know any given participant’s name or other identifying information, like their address
By looking at many LGBTQ+ people’s genetic information (DNA) along with their medical and other information, researchers hope to learn more about what factors (*e.g.*, disease exposures, lifestyle factors, genetics, discrimination, social support systems) affect diseases and conditions that more commonly affect LGBTQ+ individuals. This information could then be used to learn about how to improve people’s health
This type of research does not include studies to learn more about what role, if any, people’s genes play in their sexual orientation or gender identity. Instead, the goal of this type of research is to learn more about how to address health and disease in LGBTQ+ people

- Potential benefits
  - For interviewee; for other LGBTQ+ people
  - Reasons that *someone else* might think this research would be beneficial
- Potential risks, concerns
  - For interviewee; for other LGBTQ+ people
  - Reasons that *someone else* might think this research would be harmful/risky?
- Likelihood that this type of research would result in discovering something beneficial to interviewee, family, community, etc.
- Acceptability of researchers using SOGI data, in combination with medical and genetic data, to investigate LGBTQ+ health only
  - Factors increasing and/or decreasing acceptability
- Specific topics
- Health-related issues or topics relating to LGBTQ+ people that are especially important to study
  - Health-related issues or topics relating to LGBTQ+ people that are especially problematic, risky, or controversial to study? (i.e., research on those issues/topics would be unacceptable, inappropriate, or otherwise too risky)

**3. Genomic Research on Genetic Contributions to Sexuality/Gender**

**Table:**

Researchers want to learn more about what role, if any, genes play in sexuality and gender. They want to use blood samples from the NRP biobank to study the DNA / genetic makeup of NRP participants who identify as LGBTQ+
If their study is approved by the NRP, researchers will use special computer systems that use EHR and survey data to find which biospecimens belong to these participants, but they won’t know any given participant’s name or other identifying information, like their address
By looking at the genes of many LGBT + people, researchers hope to learn more about the role of genetics in sexuality and gender. In other words, researchers want to find out the extent to which people’s sexuality or gender is influenced by their genes

- Potential benefits
  - For interviewee; for other LGBTQ+ people
  - Reasons that someone else might think this research would be beneficial
- Potential risks, concerns
  - For interviewee; for other LGBTQ+ people
  - Reasons that someone else might think this research would be harmful/risky?
- Potential Results
  - Scenario A: Research develops evidence that genetic makeup does affect sexuality and/or gender
    o Internal effects: Personhood, identity, personal meaning, etc.
    o External effects: Socio-cultural, financial, familial, political, etc.
  - Potential Results – Scenario B: Research develops evidence that genetic makeup does not affect sexuality and/or gender
    o Internal effects: Personhood, identity, personal meaning, etc.
    o External effects: Socio-cultural, financial, familial, political, etc.
  - Potential Results – Scenario C: Research develops evidence that the genetic variation that contributes to a person’s [SO/GI] also has implications for their physical / mental health
    o Internal effects: Personhood, identity, personal meaning, etc.
    o External effects: Socio-cultural, financial, familial, political, etc.
- Likelihood that this type of research would result in discovering something beneficial to interviewee, family, community, etc.
- Acceptability of researchers using SOGI data, in combination with medical and genetic data, to investigate genetic contributions to sexuality and/or gender
  - Factors increasing and/or decreasing acceptability

**GENERAL**

- Key issues
  - Most important things that LGBTQIA + individuals should consider when deciding whether or not to participate in genetic research studies that might use SOGI data
  - Most important things that *researchers* should consider when designing and conducting genetic research studies that use SOGI data
- Likelihood of personally participating in NRP

### Appendix A3. Consolidated Criteria for Reporting Qualitative Studies (COREQ)

**Table:**

**RESEARCH TEAM AND REFLEXIVITY**	
**Personal characteristics**	
1. interviewer: Which authors conducted the interviews?	The interviews were conducted by Catherine Hammack-Aviran (author) and Carolyn Diehl (author) under the leadership of the Principal Investigator Ellen W. Clayton (author)
2. credentials: What were the researcher’s credentials?	Ellen W. Clayton, JD, MD (author); *Principal Investigator*; heterosexual cisgender woman; law, bioethics, genomics, policy Lea K. Davis, PhD (author); *Associate Professor of Biomedical Informatics, Associate Professor of Psychiatry, Associate Professor of Molecular Physiology and Biophysics*; heterosexual cisgender woman; human genetics/genomics, polygenic inheritance, statistical genomics Carolyn Diehl, MS (author); *Center for Biomedical Ethics & Society, Program Manager*; heterosexual cisgender woman; qualitative research Ayden Eilmus, BA (author); *Research Assistant*; heterosexual cisgender woman; anthropology, medicine, health, and society, philosophy Gilbert Gonzales, PhD, MHA (author); *Department of Medicine, Health and Society, Assistant Professor*; gay cisgender man; LGBTQ+ health, health disparities, population health Keanan Gabriel Gottlieb, BA (author); *Health Services Research Analyst, Vanderbilt Program for LGBTQ Health*; queer transgender man; LGBTQ+ health Catherine Hammack-Aviran, MA, JD [CHA] (author); *Center for Biomedical Ethics & Society Core Faculty*, *Associate in Health Policy*; lesbian cisgender woman; law, bioethics, qualitative research
3. occupation: What was their occupation at the time of the study?	
4. gender identity and sexual orientation:	
5. experience and training: What experience or training did the researcher have?	CHA is a law and bioethics scholar with over fifteen years of experience in empirical investigations, including qualitative research and community engagement. She has published over 25 articles and book chapters regarding ethical, legal, and social issues in health and biomedical research, and serves on several national committees and working groups on research ethics GG is interdisciplinary health services researcher with expertise in sexual and gender (SGM) minority health, health policy, health disparities, and population health. He has published over 30 peer-reviewed studies examining health outcomes, access to care, and health services utilization among lesbian, gay, bisexual, and transgender (LGBT) populations AE is a recent graduate of Vanderbilt University where she focused on medical anthropology and philosophy. She has published articles on representations of genomics in film and television CD is a program manager with experience in recruiting research participants and in coding, analyzing, and managing qualitative research data LKD is a statistical geneticist whose work focuses on neurodiversity in human populations. She is devoted to advocating on issues of social justice including LGBTQ health disparities and was the senior author of the position paper by Polderman, et al., on behalf of the International Gender Diversity Genomics Consortium KGG is a research analyst, educator, patient navigator, and consultant in the Program for LGBTQ Health and was instrumental in community outreach EWC has been exploring the ethical, legal, and social implications of genomics research, its clinical applications, and social impact for over 40 years, using a wide array of methods, including qualitative research
**Relationship with participants**	
6. relationship established: Was a relationship established prior to study commencement?	No relationship was established between an interviewee and interviewer prior to study commencement. In the few instances in which an eligible participant knew or may have known an interviewer in a personal or professional capacity outside of the research context, the participant was informed of the potential privacy considerations and was assigned to the *other* interviewer; additional measures were taken to ensure that interviewers could not directly or indirectly deduce or assume the identity of de-identified data
7. participant knowledge of the interviewer: What did the participants know about the researcher? *(e.g., personal goals, reasons for doing the research)*	Prospective participants were provided with information about the funding source, the overall goals of the study, and the specific goals of the interview (see consent information)
8. interviewer characteristics: What characteristics were reported about the interviewer/facilitator? *(e.g., bias, assumptions, reasons and interests in the research topic)*	Some interviewees were informed or otherwise aware of their interviewer’s sexual orientation and/or gender identity in the course of the conversation. Both interviewers are cisgender; one interviewer openly identifies as LGBTQ+
**Study design**	
**Theoretical framework**	
9. methodological orientation and theory: What methodological orientation was stated to underpin the study? *(e.g., grounded theory, discourse analysis, ethnography, phenomenology, content analysis)*	We used an over-arching grounded theory research methodology. Within the overall framework, we employed an applied thematic analysis (including constant comparative analysis) to identify and refine meaningful categories Additional details are presented in COREQ Section 25, below
**Participant selection**	
10. sampling: How were participants selected? *(e.g., purposive, convenience, consecutive, snowball)*	Purposive and referral sampling, as described under *Methods: Participants*
11. method of approach: How were participants approached? *(e.g. face-to-face, telephone, mail, email)*	Prospective participants were informed of the opportunity to participate in this study via email initially distributed by local LGBTQ+ organizations (*see acknowledgments*). The study was also advertised on popular social media platforms by the Vanderbilt Program for LGBTQ+ Health, and multiple participants reported having learned of the study through word-of-mouth
12. sample size: How many participants were in the study?	n = 31
13. non-participation: How many people refused to participate or dropped out? Reasons?	Among the individuals who contacted us to express interest in participating: • 5 were not eligible • 0 were eligible but declined to participate ◦ 0 were unavailable (*e.g.*, too busy) ◦ 0 did not specify reason • 5 agreed but failed to respond to our attempts to schedule an interview • 1 scheduled an interview but did not participate (*i.e.*, “no-shows”) No individual failed to complete an interview in progress (*i.e.*, no one dropped out), and no completed interviews were omitted from the dataset
**Setting**	
14. setting of data collection: Where was the data collected? (e*.g., home, clinic, workplace*)	Interviews were conducted by telephone
15. presence of non-participants: Was anyone else present besides the participants and researchers?	No. Importantly, we specifically asked participants to confirm at the start of the interview that their environment was safe, private, and conducive to transparent conversation regarding sexuality and gender
16. description of sample: What are the important characteristics of the sample? *(e.g., demographic data, date)*	The sample is described in detail under *Methods: Participants* and under *Results: Participant Characteristics*)
**DATA COLLECTION**	
17. interview guide: Were questions, prompts, guides provided by the authors? Was it pilot tested?	The interview questions and prompts associated with the data reported here are provided under *Methods: Instrument Development*; the instrument is available upon request. The interview guide, including the hypothetical scenarios, was developed via stakeholder engagement and pilot tested with 3 individuals representing a range of LGBTQ+ identities, racial/ethnic backgrounds, and geographic locations
18. repeat interviews: Were repeat interviews carried out? If yes, how many?	No interviews were repeated
19. audio/visual recording: Did the research use audio or visual recording to collect the data?	All interviews were digitally audio-recorded per each participant’s verbal permission
20. field notes: Were field notes made during and/or after the interview or focus group?	Yes; interviewers took handwritten notes directly onto interview materials designated for each participant throughout the interview, as well as additional post-interview contextual notes when relevant
21. duration: What was the duration of the interviews?	Interviews ranged from 30—57 min in length. On average, each interview lasted approximately 45 min
22. data saturation: Was data saturation discussed?	All transcripts were reviewed in their entirety during team-based codebook development for the purpose of generating codes to capture all primary themes (with the goal of capturing all secondary and tertiary themes). After independent application of the finalized codebook to 10 randomly-selected transcripts, no additional themes were identified, suggesting saturation
23. transcripts returned: Were transcripts returned to participants for comment and/or correction?	No
**Analysis and findings**	
**Data analysis**	
24. number of data coders: How many data coders coded the data?	Two (2) [CHA, CD]
25. description of the coding tree: Did authors provide a description of the coding tree?	Major and minor themes are identified within distinct headings and subheadings
26. derivation of themes: Were themes identified in advance or derived from the data?	Themes were derived from the data Specifically, several a priori codes were identified through systematic instrument development; e.g., overall acceptability of each research scenario) To identify emergent content and structural themes, all members of our interdisciplinary team—with expertise in law, bioethics (including empirical bioethics), genomics, LGBTQ+ health, trans health, health policy, and qualitative research–reviewed at least five (5) transcripts. Each transcript (31) was assigned to at least two team members, and every transcript was reviewed by at least one of three team members who self-identify as LGBTQ+ . Each team member independently reviewed transcripts, identifying important and/or common themes and compiling an initial list of potential structural and thematic codes. The primary coder [CHA] compiled all potential codes, and the team converged multiple times to revise and refine code definitions, inclusion/exclusion code application criteria, and examples of code applications to representative data. Primary and secondary structural codes were then applied to all 31 transcripts by CHA (~ 65%) and CD (~ 35%). Given the extensive complexities and nuances in the data, the primary coder [CHA] (self-identifying LGBTQ+ person) applied content codes to all transcripts; the secondary coder [CD] (not LGBTQ+ person) then independently reviewed all content code applications to confirm accuracy and identify errors, inconsistencies, or discrepancies, which were then resolved in discussion with the primary coder and larger team, when applicable
27. software: What software, if applicable, was used to manage the data?	NVivo 12 (2018)
28. participant checking: Did participants provide feedback on the findings?	No
Reporting	
29. quotations presented: Were participant quotations presented to illustrate the themes / findings? Was each quotation identified? *(e.g., participant number)*	Participant quotations are presented and identified by participant pseudo-identifier
30. data and findings consistent: Was there consistency between the data presented and the findings?	Our manuscript integrates extensive use of direct quotes to provide evidence for each conclusion drawn
31. clarity of major themes: Were major themes clearly presented in the findings?	Major themes are clearly identified within distinct headings and subheadings
32. clarity of minor themes: Is there a description of diverse cases or discussion of minor themes?	There is substantial discussion of themes within each subheading, including diverse cases and minority opinions
